# Insulin-like growth factor II mRNA binding protein 3 (IMP3) is overexpressed in prostate cancer and correlates with higher Gleason scores

**DOI:** 10.1186/1471-2407-10-341

**Published:** 2010-06-30

**Authors:** Kristian Ikenberg, Florian R Fritzsche, Ursina Zuerrer-Haerdi, Irina Hofmann, Thomas Hermanns, Helge Seifert, Michael Müntener, Maurizio Provenzano, Tullio Sulser, Silvia Behnke, Josefine Gerhardt, Ashkan Mortezavi, Peter Wild, Ferdinand Hofstädter, Maximilian Burger, Holger Moch, Glen Kristiansen

**Affiliations:** 1 Institute of Surgical Pathology, University Hospital Zurich, Zurich, Switzerland; 2 Department of Urology, University Hospital Zurich, Zurich, Switzerland; 3 Institute of Pathology, University of Regensburg, Regensburg, Germany; 4 Department of Urology, Caritas-St. Josef Medical Center, University of Regensburg, Regensburg, Germany

## Abstract

**Background:**

The oncofetal protein insulin-like growth factor II mRNA binding protein 3 (IMP3) is an important factor for cell-migration and adhesion in malignancies. Recent studies have shown a remarkable overexpression of IMP3 in different human malignant neoplasms and also revealed it as an important prognostic marker in some tumor entities. To our knowledge, IMP3 expression has not been investigated in prostate carcinomas so far.

**Methods:**

Immunohistochemical stainings for IMP3 were performed on tissue microarray (TMA) organized samples from 507 patients: 31 normal prostate tissues, 425 primary carcinomas and 51 prostate cancer metastases or castration-resistant prostate cancers (CRPC). IMP3 immunoreactivity was semiquantitatively scored and correlated with clinical-pathologic parameters including survival.

**Results:**

IMP3 is significantly stronger expressed in prostate carcinomas compared to normal prostate tissues (p < 0.0001), but did not show significant correlation with the pT-stage, the proliferation index (MIB1), preoperative serum PSA level and the margin status. Only a weak and slightly significant correlation was found with the Gleason score and IMP3 expression failed to show prognostic significance in clinico-pathological correlation-analyses.

**Conclusions:**

Although IMP3 is overexpressed in a significant proportion of prostate cancer cases, which might be of importance for novel therapeutic approaches, it does not appear to possess any immediate diagnostic or prognostic value, limiting its potential as a tissue biomarker for prostate cancer. These results might be corroborated by the fact, that two independent tumor cohorts were separately reviewed.

## Background

Insulin-like growth factor II mRNA binding protein 3 (IMP3), an oncofetal protein and member of the insulin-like growth factor II mRNA binding protein family, has recently raised attention since it appears to play an important role in cell-migration and adhesion in various malignant neoplasms [1]. It functions in RNA shuttling and translational control: to date three members of this family are known: IMP1, IMP2 and IMP3 [2,3]. The human IMP3 gene is located at chromosome 7p15 with an identical sequence to that of KOC (KH domain containing protein overexpressed in cancer) and shows an overall sequence identitiy of 59% with other mRNA binding family members [4].

Physiologically, IMP3 is commonly expressed during embryogenesis in mouse and human organs, but rarely in adult tissue [5]. Current studies reveal a remarkable re-expression of IMP3 in different human malignancies, e.g. in ovarian cancer, non-small cell lung cancer, malignant melanoma, osteosarcoma, pancreatic ductal adenocarcinoma, melanoma, metastatic melanoma, colorectal adenocarcinoma, urothelial tumors, extrapulmonary small cell carcinoma, endometrial carcinoma, malignant follicular pattern thyroid lesions, invasive mammary carcinoma, esophageal adenocarcinoma, adenocarcinoma on endoscopic bile duct biopsy/bile duct carcinoma and in high-grade dysplasia in the extrahepatic biliary tract [6-23].

As the most common malignant neoplasm in men, prostate cancer is the second most common cause of tumor related deaths in the United States [24]. Although prostate cancer is commonly a slowly progressing disease that might not become clinically apparent during the patient's lifetime, a certain proportion of cases does take a more serious course associated with a poor clinical outcome [25]. Conventional prognostic factors such as Gleason score, preoperative PSA levels or ratio of involved biopsies only insufficiently predict patient outcome for currently available therapies. They are even more limited in identifying insignificant prostate cancer, i.e. cancer that might be left untreated without shortening the patients life expectancy but sparing him the morbidity of unwarranted treatment. Therefore, further efforts to find new diagnostic pathways and therapeutic options are urgently demanded to optimise patient management [26].

In this study we analysed by immunohistochemistry the protein expression profile of IMP3 in benign prostate tissue, primary prostate cancer, castration resistant prostate cancer (CRPC) and prostate cancer metastases and correlated IMP3 expression to clinico-pathological parameters including biochemical recurrence times.

## Methods

### Patients

Tissue samples from 507 patients were enclosed in this study: 83.8% (n = 425) primary carcinoma following radical prostatectomy in the department of Urology of the University Hospital of Zurich (USZ) and the department of Urology of the University Hospital of Regensburg, 10.1% (n = 51) prostate cancer metastases or castration-resistant prostate cancer (CRPC) and 6.1% (n = 31) non-malignant/normal prostate tissue. For Zurich, the median follow up time of the patients was 95 months (0 to 167 months), median age 66 (46 to 95 years). For Regensburg, the median follow up time of the patients was 63 months (0 to 111 months), median age 63 (47 to 76 years). None of the patients received hormonal therapy or chemotherapy prior to surgery.

### Tissue Microarray (TMA)

For the construction of the tissue microarray (TMA) fomalin fixed and paraffin embedded tissue was used as described previously [27]. Briefly, from tissue sample from each patient one core of 0.6 mm diameter was punched out from the donor block and transfered to the recipient (TMA) block. Each tumor was represented by a single but representative core. To exclude the potential influence of molecular field effects, normal tissue was sampled from transurethral resections of patients with benign hyperplasia of the prostate and unremarkable PSA levels.

### Immunohistochemistry/Evaluation of the staining pattern

For immunohistochemical staining freshly cut 3 μm thick sections of the TMA block were mounted on superfrost slides (Menzel Gläser, Braunschweig, Germany). For IMP3 detection, staining were performed on a BondMax automated staining system (Vision BioSystems Ltd., Newcastle upon Tyne, United Kingdom) with a monoclonal mouse antibody (clone 69.1, dilution 1:100, DAKO, Glostrup, Denmark). After epitope retrieval (H2-Buffer, Vision BioSystems Ltd., Newcastle upon Tyne, United Kingdom) primary antibody was detected by Refine DAB method (Vision BioSystems Ltd., Newcastle upon Tyne, United Kingdom). Slides were counterstained with hematoxylin prior to dehydration and coverslipping.

Since IMP3 protein is relatively homogeneous expressed on prostate cancer, staining intensity (exclusively cytoplasmatic staining pattern was evaluated) was assigned to a semiquantitive, four-tired score. In cases with heterogeneity, the predominant staining intensity (> 80%) was counted: (0) negative, + (1) weak, ++ (2) moderate, +++ (3) strong. Evaluation was done by KI, GK, UZ and FF simultaneously at a multiheaded microscope.

### Statistics

Statistical analyses were performed with SPSS, Version 17, according to Spearman and with Linear-by-linear association to compare the IMP3 expression pattern with clinico-pathologic parameters. Analysis of biochemical recurrence times was performed according to Kaplan Meier with log rank test.

## Results

### Expression of the IMP3 protein in prostate tissues

In samples from non neoplastic prostate tissue and from benign prostate hyperplasia, no remarkable IMP3 protein expression was found (n = 31, 100% negative).

**Figure 1:**
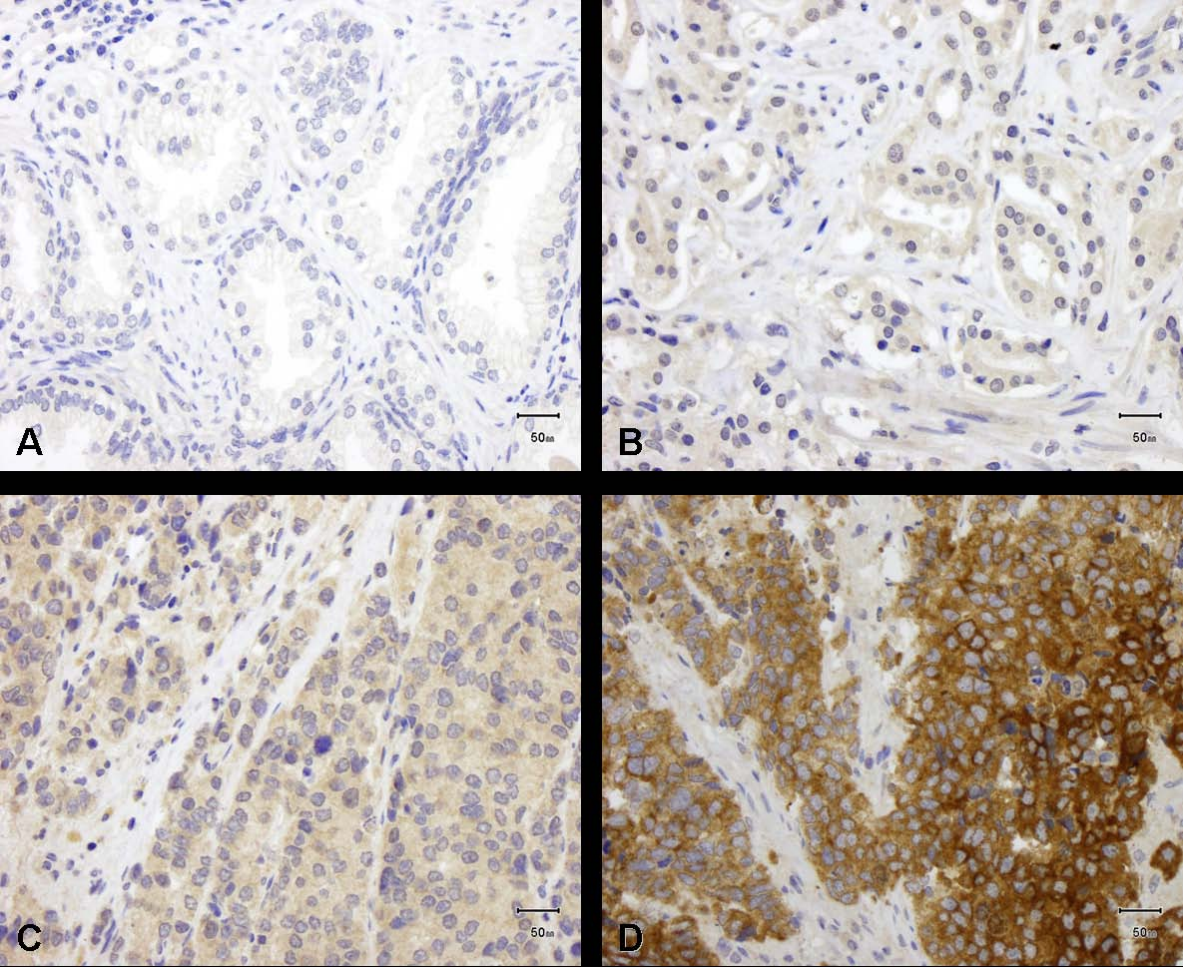
**IMP3 expression in benign prostate tissue and prostate carcinoma**. (A) No or weak IMP3 expression in benign prostate tissue; (B-D) IMP3 expression in primary prostate carcinoma: (B) weak, (C) moderate and (D) strong expression.

The primary prostate carcinomas (n = 425) 16.7% (n = 71) showed no IMP3 expression, 69.2% (n = 294) did show weak staining, 13.4% (n = 57) moderate and 0.7% (n = 3) strong staining for IMP3 (median = 1; Figure 1).

51 samples from lymphnode-and organ metastases as well as from cases with castration-resistant prostate cancer disease were analysed: 25.5% (n = 13) showed no IMP3 expression, 58.8% (n = 30) a weak expression, 5.9% (n = 3) moderate and 9.8% (n = 5) strong IMP3 expression level (median = 1).

The difference of IMP3 immunoreactivity between normal and tumor tissues was highly significant (p < 0.001).

### Correlation of IMP3 expression with clinico-pathological factors

In a nonparametric Spearman rank correlation analysis, the expression of IMP3 did show a minor, but weak significant correlation with the Gleason score (correlation coefficient (CC) = 0.1, p = 0.04). No significant association of IMP3 was observed with pT-stage (CC = 0.04, p = 0.412), the proliferation index (MIB1) (CC = 0.060, p = 0.377), preoperative serum PSA level (CC = 0.061, p = 0.213) and the margin status (R0 *vs*. R1) (CC = 0.089, p = 0.068). Additional cross table analysis validated these results (Table 1).

**Table 1:** IMP3 related to clinico-pathological parameters of prostate cancer

Variable	Patients (n)	Number of patients (IMP-Expression) (%)				p-Value (linear by linear)
		0	1	2	3	
**pT-status**						0.5
**pT2**	213	46 (21.6%)	131 (61.5%)	34 (16%)	2 (0.9%)	
**pT3&4**	212	25 (11.8%)	163 (76.9%)	23 (10.8%)	1 (0.5%)	
**Gleason score**						0.048
**5-6**	129	29 (22.5%)	85 (65.9%)	14 (10.9%)	1 (0.8%)	
**7**	182	28 (15.4%)	127 (69.8%)	26 (14.3%)	1 (0.5%)	
**8-9**	114	14 (12.3%)	82 (71.9%)	17 (14.9%)	1 (0.9%)	
**Margin status**						0.115
**R0**	262	54 (20.6%)	171 (65.3%)	34 (13%)	3 (1.1%)	
**R1**	163	17 (10.4%)	123 (75.5%)	23 (14.1%)	0 (0%)	
**PSA (pre-OP)**						0.21
**< 10 ng/ml**	192	37 (19.3%)	130 (67.7%)	24 (12.5%)	1 (0.5%)	
**≥10 ng/ml**	233	34 (14.6%)	164 (70.4%)	33 (14.2%)	2 (0.9%)	
**Age**						0.693
**≤64 y**	230	33 (14.3%)	168 (73%)	27 (11.7%)	2 (0.9%)	
**> 64 y**	195	38 (19.5%)	126 (64.6%)	30 (15.4%)	1 (0.5%)	

Distribution of IMP3 expression in primary prostate cancer according to clinico-pathological parameters

### Survival analysis

**Figure 2:**
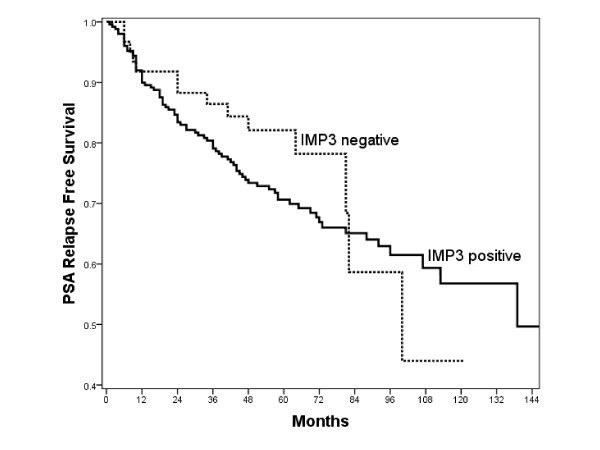
**Kaplan Meier survival analysis**. Kaplan-Meier survival analysis and IMP3 expression (negative *vs*. positive) in patients with primary prostate carcinoma (p = 0.343).

In an univariate Cox-analysis for primary carcinomas, Gleason-score (5-6 *vs*. 7 *vs*. 8-9), pT-status (pT2 *vs*. pT3/4), pre OP PSA (< 10 ng/ml *vs*. ≥10 ng/ml) and margin-status (R0 *vs*. R1) were confirmed as significant prognostic factors for PSA free survival times whereas patients age (≤64y vs. > 64y) and IMP3 expression (0/1/2/3) were not significant factors (Table 2). Also, in a Kaplan-Meier survival analysis there was a non significant prognostic value for IMP3 expression (negative *vs*. positive) (p = 0.343), although IMP3 positive cases showed a slightly more unfavourable course (Figure 2). If analysed separately, in both cohorts from Zurich (USZ) and Regensburg IMP3 expression was not a significant factor: Zurich RR (Exp(B)) = 1.444, p-Value = 0.102; Regensburg RR (Exp(B)) = 1.022, p-Value = 0.932.

**Table 2:** Univariate Cox analysis of PSA recurrence free survival times

Parameter		RR (Exp (B))	p-Value
**Gleason-Score**	**5-6/7/8-9**	1.713	< 0.001
**pT-status**	**pT2/pT3&4**	2.387	< 0.001
**pre OP PSA**	**< 10 ng/ml/≥10 ng/ml**	1.95	0.003
**Age**	**≤64y/> 64y**	1.169	0.449
**Margin status**	**R0/R1**	2.204	< 0.001
**IMP3**	**0/1/2/3**	1.181	0.332

## Discussion

An accurate and early diagnosis is essential for efficient management of prostate cancer [24,25]. Therefore, to complement improvements in the clinical management, substantial progress in the diagnostic pathway of prostate cancer is urgently needed [26].

The oncofetal protein IMP3 plays an important role in translational control of Insulin-like Growth Factor II leader-3 mRNA during proliferation and in cell-adhesion [1,3,28] and it regulates, among others, CD24 and CD166, both of which we have identified as prognostic markers for prostate cancer in earlier studies [1,29]. Therefore we had expected a prognostic value of IMP3 in prostate cancer, as has already been shown in other malignancies [6-23]. In addition, a first phase I clinical trial with an anti-IMP3 immunotherapy in non-small cell lung cancer showed a high level of safety and so potentially offers a new therapeutic option for other malignancies as well [30]. This demonstrates that IMP3 has not only a high diagnostic potential, but is also a very promising target for therapy, suggesting further studies into this member of the insulin-like growth factor II mRNA binding protein family are warranted.

Our results show, that IMP3 is only rarely expressed in benign prostate tissue whereas it shows a significantly but only slightly higher expression level in malignant prostate tissue. The diagnostic use of IMP3 in difficult or doubtful lesions found at biopsy cannot be recommended, since most cases show only a weak IMP expression, which can - in a diagnostic real life situation - be troublesome to differentiate from background. Only approximately 14.1% (n = 60) of cases showed moderate to strong IMP3 expression, a rate too low to recommend the use of IMP3 as a diagnostic marker of malignancy. Although higher rates of IMP3 expression where seen in cases with higher Gleason scores, higher pre-OP PSA level and higher proliferative fractions, still no significant prognostic relevance for IMP3 could be demonstrated (Table 2). The IMP3 expression (negative *vs*. positive) in a Kaplan-Meier survival analysis appears to possess a minor prognostic value at first sight, but this fails to be significant (p = 0.343) and can at best be interpreted as a very loose trend. One might argue that this lack of prognostic significance we found could be an idiosyncrasy of this tumor cohort. In this study two different patient/tumor cohorts were analysed, so it is unlikely, that a larger cohort with even longer follow up times would demonstrate a prognostic value of IMP3. We cannot exclude a possible minor prognostic value of IMP3, but on the basis of our data we assume that IMP3 is very unlikely to represent a strong prognostic biomarker in prostate cancer.

## Conclusions

Although IMP3 is overexpressed in prostate cancer, preferentially of higher Gleason scores, it does not have the same outstanding diagnostic and prognostic value for prostate cancer, as it has for other malignancies.

## Competing interests

The authors declare that they have no competing interests.

## Authors' contributions

Analysis of the TMA was performed by KI, FF, UZ and GK. The manuscript was written by KI and critically revised by GK, FF, JG, AM, PW and HM. TMA data were provided by GK. Statistical analyses were performed by GK, KI, FF and IH. SB evaluated immunostaining and performed data analysis. TH, HS, MM, MP, TS, FH, MB provided clinical samples and provided clinical follow-up data. All authors read and approved the final manuscript.

## Pre-publication history

The pre-publication history for this paper can be accessed here:

http://www.biomedcentral.com/1471-2407/10/341/prepub
